# Compassionate and respectful maternity care during facility based child birth and women’s intent to use maternity service in Bahir Dar, Ethiopia

**DOI:** 10.1186/s12884-018-1909-8

**Published:** 2018-07-09

**Authors:** Biresaw Wassihun, Shegaw Zeleke

**Affiliations:** 1 College of Medicine and Health Sciences, Arba Minch University, Arba Minch, Ethiopia; 2 Colleges of Medicine and Health Sciences, Debre Tabor University, Debre Tabor, Ethiopia

**Keywords:** Compassionate, Respectful, Disrespect, Abuse, Child birth, Ethiopia

## Abstract

**Background:**

Compassionate and respectful maternity care is one of the most important facilitating factors to increase access to skilled maternity care. Disrespect and abuse is a violation of human rights and is the main hindering factor preventing skilled birth utilization versus other more commonly recognized deterrents such as financial and geographical obstacles.

**Methods:**

Institution based cross-sectional study design was conducted. A structured and pre-tested interviewer administered questionnaire was used to collect the data from 284 study participants. Study participant were selected using a systematic random sampling technique by allocating a proportion to each health facility. The data were entered with Epi data version 3.1statistical software and exported to Statistical Package for Social Sciences version 22.0 for further analysis. Both bivariate and multivariate logistic regression analysis were performed to identify associated factors. *P* values < 0.05 with 95% confidence level were used to declare statistical significance.

**Result:**

A total of 284 respondents participated in the study with a response rate of 100%.The overall prevalence of respectful maternity care experienced was 57%.The multivariable analysis indicated that respondents who live in a rural area [AOR = 6.49(95%CI; 2.59, 16.21)], experience a caesarian birth [AOR = 4.52(95%CI; 1.64, 12.42)], have complications during delivery [AOR = 2.38(95%CI; 1.28, 4.45)] and future intention to use health facility [AOR = 3.57(95%CI; 1.81, 7.07)] were some of the factors associated with experiencing disrespect and abuse.

**Conclusion:**

This study showed a high prevalence of disrespect and abuse during facility child birth in Bahir Dar town, Ethiopia as compared to previous literature. Being from rural area, having complications during delivery and mothers who gave birth through caesarian section were more likely to be exposed to disrespect and abuse than other women. Mistreatment of mothers during facility child birth is a health facility failure, a violation of women’s rights and a notable barrier for institutional delivery.

## Backgroud

Child birth is a special moment for parents, families and communities but can also be a time of intense vulnerability for women to be exposed to disrespectful and abusive behavior while at the health facilities. If mothers do not receive what they perceive as compassionate and respectful maternity care during facility based child birth their intention to use facility based maternity services will be reduced [1].

The concept of “safe motherhood” is usually restricted to physical safety, but childbearing is also an important passage from woman to mother, with deep personal and cultural significance for a woman and her family. Because motherhood is specific to women, issues of gender equity and gender violence are also at the core of maternity care [2].

In recent years advocacy for skilled birth attendance has increased and has become the single most important intervention suggested to decrease maternal mortality and morbidity. However, client satisfaction with such care, which is an important element of quality care, which has the potential to influences effectiveness of care, has not been given enough attention [2, 3].

Problems related to maternal provider behaviors and attitudes are a major barrier when compared to any other geographical or financial barriers to utilization of skilled childbirth care [4].

Advancing compassionate and respectful maternity care is critical in order to increase facility based child birth and ensure effective implementation of women’s rights and women-centered approaches in maternal health services. In fact efforts to increase the use of facility based maternity care service in low resources countries are unlikely to achieve desired gains without improving the quality of care and focusing on women’s experience of care [5].

Despite advances in maternal health outcomes, ensuring that women have skilled and respectful care during delivery remains a challenge. In many countries, women are mistreated when delivering in health facilities and unable to make choices or follow practices that allow them to be in control of their own experience [6]. In addition, health systems are underequipped and health workers are not satisfied due to inadequate pay, lack of infrastructure, or insufficient staff and supplies; staff may also not receive guidance, support or supervision facilitating the provision of respectful maternity care [7].

According to the WHO statement on the prevention and elimination of disrespect and abuse of women during facility-based childbirth and the White Ribbon Alliance charter, respectful maternal care refers to “the right of every woman to the highest attainable standard of health, which includes the right to dignified, respectful health care at all health systems around the world of childbearing woman throughout her pregnancy, during labour and delivery, and post natal period” [1, 8]. Maternal morbidity and mortality has continued to be a public health problem globally as evidenced by high maternal morbidity and mortality [9]. Globally in 2015, an estimated 303,000 women have lost their lives due to easily preventable pregnancy and childbirth related complications, 99% of which were in low income countries. In this figure, sub- Saharan Africa alone was accountable for 66% of the deaths [10].

Ethiopia has one of the highest maternal mortality rates globally (MMR) of 412 maternal deaths per 100,000 live births [11].

In countries where more than 80% of births are attended by trained health personnel, the rate of maternal mortality is less than 200 per 100,000 live births. Thus one of the strategies designed to decrease maternal morbidity and mortality was to increase maternity up take with skilled health care providers [9, 12, 13].

The proportion of women who are giving birth at health institutions with trained birth attendants in Ethiopia is not more than 28%. [11] . Multiple studies have examined reasons for failing to use skilled services during delivery. However, there is inadequate research linking the role of disrespect and abuse of women during facility based deliveries and decreased utilization of maternity services. Disrespect and abuse during childbirth, which is a violation of a universal human right that is due to every childbearing woman in every health system, is common throughout the world and can occur at the level of interaction between the woman and provider leading to low intake of maternity service [14–16].

Despite the problem, little research has been conducted in Ethiopia with regard to the status of disrespect and abuse during facility-based childbirth. Therefore this study aimed at assessing the status of compassionate and respectful maternity care and associated factors in health facility-based childbirth in Bahir Dar town.

## Methods

### Study area

This study was conducted in public health facilities in Bahir Dar town. Bahir Dar is located in North Western part of Ethiopia, in Amhara National Regional State, at a distance of 565 km from Addis Ababa. The total population of the town is 290,437of which 142,068 are males and 148,369 are female. In Bahir, Dare town there are 10 public health centers and two public hospitals and two private health institutions. The study was conducted in five public health facilities; four health centers and one referral hospital.

#### Study design and period

Institution based cross-sectional study was conducted from Feb 2- April 26–2017.

#### Population

Mothers who gave birth in Bahir Dar tow health facilities.

#### Sample size determination

A single proportion formula was used to estimate the sample size required for the study. The sample size calculation assumed the proportion (p) estimated level of respectful and abuse free maternity care 21.4% [17]. Adding non-response rate of 10% and considering the assumption of 95% confidence level, 5% margin of error the final sample size was 284 mothers.

#### Sampling procedure

In this study area there are ten public health centers and two public hospitals (one referral hospital and second general hospital). Four public health centers and one referral hospital was randomly selected. The allocation of the sample to health facilities was made proportionally based on the average number of clients who received childbirth services at each facility in the month preceding the data collection period. Felege-Hiwot Referral Hospital 129: Bahir Dar Health center 48: Han Health center 29: Tis-Abay Health center 45 and Shinbut Health center 33. Individual participants in each of the health facilities were selected by systematic random sampling during the data collection period until the required sample size at each health facility was obtained. The sampling interval k = 3 was calculated by dividing the source population to the total sample size and this interval was used in all health facility to select study participants. The first client was selected by simple random sampling among the first three maternity care users in the sampling frame.

#### Operational definition

##### Respectful maternity care (RMC)

A universal human right that is due to every childbearing woman in every health system around the world in which the maternity care is expanded beyond the prevention of morbidity or mortality to encompass respect for women’s basic human rights, including respect for women’s autonomy, dignity, feelings, choices, and preferences, such as having a companion wherever possible [18].

### Data collection tool

The data collection method that was used in this study was face to face interviews using a structured questionnaire. The English version questionnaire was translated into local language Amharic to obtain data from the study participants and to ensure clarity of its content. Then the Amharic version was transcribed back to English version to check for consistency. It was prepared by the principal investigator based on literature reviews, and from Maternal and Child Health Integrated Program (MCHIP) as part of their respectful maternity care tool kit [3]. The questionnaire was designed to obtain information on socio demographic-characteristics and factors associated with disrespect and abuse. The instrument was pretested for its reliability. The content validity of the questionnaire was reviewed by experienced public health professionals.

### Data collection procedure and quality control

Before actual data collection occurred two day training was provided for data collectors and the supervisor about techniques of data collection and briefed on each questions included in the data collection tool. Pretest was done on 10% (28) of mothers receiving care in a health center that was not included in the study prior to the actual study period. After pre-testing the questionnaire, Cronbatch’s Alpha was calculated by using SPSS window version 22.0 to test internal consistency (reliability) of the item and Cronbatch’s Alpha greater than 0.7 was considered as reliable. Data were collected by trained midwives and nurses. During data collection regular supervision was done by the supervisors.

### Data processing and analysis

First the collected data were checked manually for completion and any incomplete or misfiled questions. The data were cleaned and stored for consistency, entered into Epi Data version 3.1 software then exported to statistical package for social sciences (SPSS) version 22.0 software for analysis. The accuracy of the data entry was checked by double data entry. Any errors identified during data entry were corrected by reviewing the original completed questionnaire. Descriptive statistics were done and presented using tables and figures. Initially, bivariate logistic regression was carried out to see the association of each of the independent variables with the outcome variable. Thereafter, the multivariable logistic regression method was used. The variables that were not significant in the bivariate logistic regression were not considered in the multiple regression analysis. P- Value of < 0.05 and 95% confidence level was used as a difference of statistical significance. Finally, results were compiled and presented using tables, graphs, and text.

## Results

### Socio-demographic characteristics of respondent

From a total of 284 respondents who were invited for interview all consented to participate in the study giving a response rate of (100%). Mean age of the respondents was 28.3 (SD ± 6.5) years with a minimum and maximum age of 15 and 46 respectively. Almost all of the study participants 230 (81.0%) were from the Amhara ethnic group and 211 (74.3%) were Orthodox Christianity religion followers. Regarding marital status of the mother, 259 (91.2%) of them were married. Out of the total respondents 145 (51.1%) of them had a monthly family income < 3000 Ethiopian birr. Almost all of the respondents 250 (88.0%) were not able to pay for any medical services. Of the total respondents 235 (82.7) were urban residents (Table 1).

**Table 1:** Socio demographic characteristics of respondents in Bahir Dar town public health facility, North West Ethiopia, 2017 (*n* = 284)

Variables	Frequency	Percentage
Age		
15–19	4	1.4
20–24	130	45.8
25–29	28	9.9
30–34	91	32.0
35 and above	31	10.9
Occupation		
House wife	57	20.1
Government employee	182	64.1
Private business	45	15.8
Ethnicity		
Amhara	230	81.0
Oromo	14	4.9
Awi	25	8.8
Tigira	12	4.2
Others	3	1.1
Religion		
Orthodox	212	74.7
Protestant	24	8.8
Muslim	48	16.9
Marital status		
Married	259	91.2
Divorced	10	3.5
Single	11	3.9
Widowed	2	0.7
Separated	2	0.7
Mothers educational status		
Illiterate	13	4.6
Read and write	23	8.1
Elementary school (grade 1–4)	29	10.2
Secondary school (grade 5–8)	23	8.1
High school (grade 9–12)	13	4.6
Diploma and above	183	64.5
Family monthly income		
< 3000	145	51.1
≥ 3000	139	48.9
Median income 3000		
Residence		
Urban	235	82.7
Rural	49	17.3

### Obstetric history of respondents

**Fig. 1:**
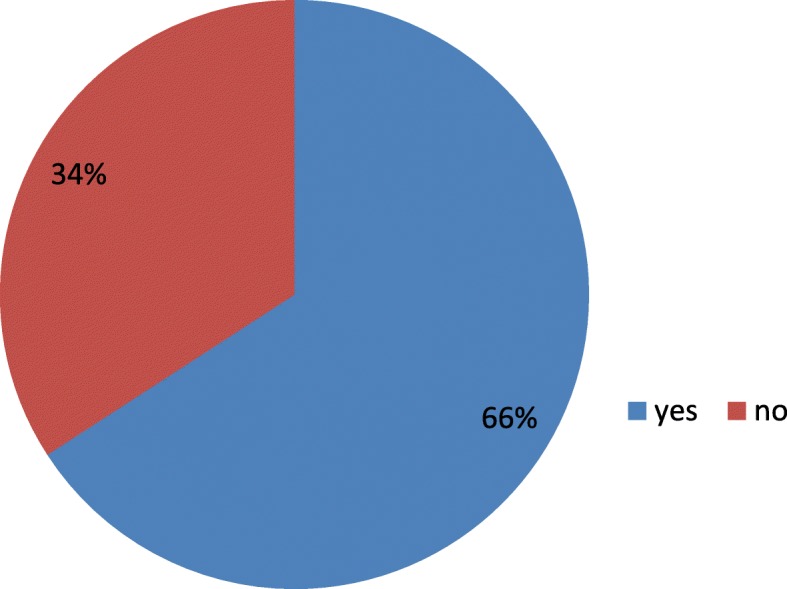
Intention of respondents to give birth in Bahir Dar town public health facility, North West Ethiopia, 2017

From the total of 284 respondents 267 (94.0%) had a history of antenatal care follow up. The majority of mothers 253 (89.1) were multigravida and 255 (89.8) were multipara. Almost all of the respondents 260 (91.5%) of the multiparas had previously given birth in institutions. Among respondents 151 (53.2) were gave birth vaginally and 41 (14.4) were gave birth by caesarian section. From the total respondents 97 (32.4%) had no intention of giving birth in a health facility in a future birth. The main reasons for not giving birth in a health facilities was a lack of satisfaction during their current labour and delivery 73 (25.7%) followed by home preference 19 (6.7%) (Table 2, Fig. 1).

**Table 2:** .Obstetric characteristics of respondents in Bahir Dar town public health facility, North West Ethiopia, 2017 (*n* = 284)

Variables	Frequency	Percentage
Gravidity		
Prima- gravid	31	10.9
Multi gravid	253	89.1
Parity		
Primparous	29	10.2
Multiparous	253	89.8
Pregnancy wanted/planned		
Yes	238	83.8
No	46	16.2
Antenatal care		
Yes	267	94.0
No	17	6.0
Place of Antenatal care		
Public health facility	222	78.2
Private health facility	45	16.9
History of health facility delivery		
Yes	260	91.5
No	24	8.5
Current mode of delivery		
Vaginal	151	53.2
Vacuum /forceps	92	32.4
Cesarean section	41	14.4
Sex of health care provider conducting delivery		
Male	140	50.7
Female	144	49.3
Having complication		
Yes	116	40.8
No	168	59.2
Stayed in health facility		
Yes	239	84.2
No	45	15.8
Do you have intention to give birth in health facility?		
Yes	189	66.0
No	97	34.0
If no, why?		
I am not satisfied	73	75.3
Home is better	19	19.6
No reason	5	5.2

### Category of respectful maternity care (RMC)

**Fig. 2:**
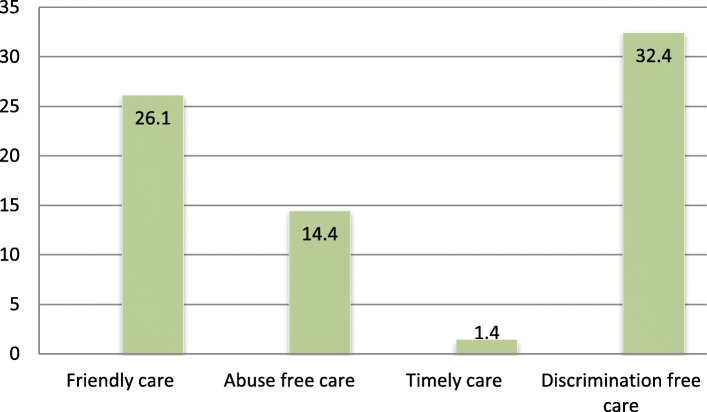
Prevalence of each category of respectful maternity care in Bahir Dar town public health facility, North West Ethiopia, 2017

The most common category of respectful maternity care identified by women in this study was providing discrimination free care 32.4% followed by providing friendly care 26.1% (Table 3, Fig. 2).

**Table 3:** Categories and types of respectfully maternity care reported by mothers during childbirth, in Bahir Dar town public health facility, North West Ethiopia, 2017 (n = 284)

Category of RMC	Types of RMC	Yes (%)	No (%)
Friendly care	I felt that health workers cared for me with a kind approach	196(69.0)	89(31.0)
	The health workers treated me in a friendly manner	194(68.3)	90(31.7)
	The HWs was talking positively about pain and relief	198(69.7)	86(30.3)
	The health worker showed his/her concern and empathy	198(69.7)	86(30.3)
	All HWs treated me with respect as an individual	195(68.7)	89(31.3)
	The HWs speak to me in a language that I can understand	194(68.3)	90(31.7)
	The health provider called me by my name	192(67.6)	92(32.4)
Abuse free care	The health worker responded to my needs whether or not I asked	198(69.7)	86(30.3)
	The health provider slapped me during delivery for different reasons	98(34.5)	186(65.5)
	The health workers shouted at me because I haven’t done what I was told to do	95(33.5)	189(66.5)
Timely care	I was kept waiting for a long time before receiving service	92(32.4)	192(67.6)
	I was allowed to practice cultural rituals in the facility	92(32.4)	192(67.6)
	Service provision was delayed due to the health facilities internal problem	89(31.3)	195(68.7)
Discrimination free care	Some of the health workers do not treated me well because of some personal attribute	92(32.4)	192(67.6)
	Some HWs insulted me and my companions due to my personal attributes	89(31.3)	195(68.7)

* RMC stands for respectful maternity care. HWs* stands for health care workers

### Prevalence of compassionate and respectful maternity care

**Fig. 3:**
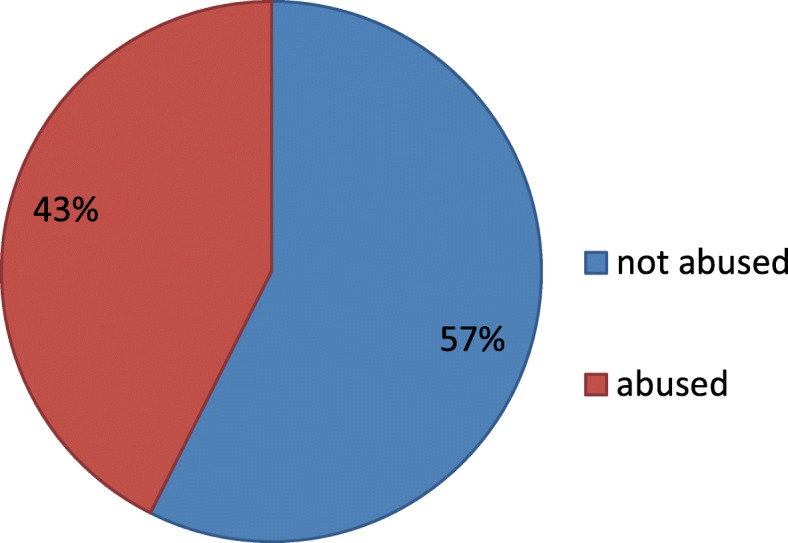
Prevalence of compassionate and respectful maternity care during facility based child birth in Bahir Dar town, public health Facilities, North West Ethiopia, 2017

Out of the 284 respondents interviewed 163(57%) experienced compassionate and respectful maternity care while 121(43%) reported having experienced at least one form of disrespect and abuse during facility based childbirth (Fig. 3).

### Factors associated with compassionate and respectful maternity care

The result of multiple logistic regression analysis showed that respondent residency, mode of delivery, time of delivery, complication during delivery, family monthly income and intention to give birth in a health facility were some of the factors associated with disrespect and abuse at *P*-value < 0.05. Respondents who were from rural area were 6.49 times more likely to report disrespect and abuse than urban residents [AOR = 6.49(95%CI; 2.59, 16.21)].Similarly respondents who gave birth through caesarian section were 4.52 times more likely to experience disrespect and abuse than those respondents who gave birth through vaginal delivery [AOR = 4.52(95%CI; 1.64, 12.42)]. Respondents who were facing complication during labor and delivery were 2.38 times more likely to experience disrespect and abuse than those respondents who did not face complications [AOR = 2.38(95%CI; 1.28, 4.45)]. Respondents who did not intend to give birth in a health facility in the future were 3.57 times more likely to have reported experiencing disrespect and abuse than those respondents planning to do so [AOR = 3.57(95%CI; 1.81, 7.07)] (Table 4).

**Table 4:** Bivariate and multivariate logistic regression analysis of disrespect and abuse and its explanatory variables in Bahir Dar town public health facility (n = 284)

Variables	Experience of D and A Yes No		COR (95%CI)	AOR (95%CI)
Age				
15–24	65	69	1	1
25–34	46	73	0.66(0.41–1.10)	0.84(0.44–1.63)
35 and above	10	21	0.50(0.22–1.15)	0.72(0.25–2.07)
Educational status				
No formal education	22	14	2.43(1.17–5.03)*	0..39(0.64–0.24)
Primary	22	30	1.13-(0.61–2.11)	0.84(0.31–1.89)
Secondary and above	77	119	1	
Family monthly income				
< 3000	76	69	2.3(1.42–3.73)*	0.90(0.46–1.75)
≥3000	45	94	1	1
Resident				
Urban	82	153	1	1
rural	39	10	7.23(3.45–15.32)*	6.49(2.59–16.21)*
Gravidity				
Prima- gravid	19	12	2.34(1.09–5.04)*	2.06(0.80–5.28)
Multi- gravid	102	151	1	1
Pregnancy planned and wanted				
Yes	84	154	1	1
No	37	9	7.54(3.47–16.37)*	0.24(0.09–0.62)
Mode of delivery				
Vaginal	63	88	1	1
Vacuum/forceps	27	65	0.58(0.33–1.00)	0.44(0.19–1.01)
Caesarian section	31	10	4.33(1.98–9.47)*	4.52(1.64–12.42)*
Time of delivery				
Day time	76	82	1.66(1.03–2.69)*	2.16(1.04–4.49)*
Night time	45	81	1	1
Facing complication during labour and delivery				
Yes	68	48	3.07(1.88–5.03)*	2.38(1.28–4.45)*
No	53	115	1	1
Intention to give birth in health facility				
Yes	52	115	1	1
No	69	28	6.39(3.72–11.02)*	3.57(1.81–7.07)*

D and A remind as disrespect and abuse,*remands as statically significant at p-value of < 0.05

## Discussion

This study investigated the prevalence and factors associated with compassionate and respectful maternity care during facility-based childbirth in Bahir Dar town at the facility level. This study revealed that the prevalence of compassionate and respectful maternity care was reported by 57% of women, with abusive maternity care reported by 43%. This proportion is higher than that in a study conducted in Kenya which showed that 20% of women reported any form of disrespect and abuse during child birth [19]. The considerable difference may be due to socio-cultural and socio-economic. It may also be due to differences in staff training and organization of healthcare. However, the proportion women reporting abuse in this study is lower than a study conducted in Enugu in Nigeria where 98% of the women have reported that they have had at least one instance of abuse during the course of childbirth [20]. The differences may also be due to the study period selected and the relatively small sample size in this study. The results of this study also show a higher level of abusive maternity care compared with data collected in Tanzania where the frequency of any abusive or disrespectful treatment during childbirth was 19% [21]. The considerable difference may be due to socio-cultural and socio-economic poupulation difference. In this study 34.5% of respondents reported that a health care provider slapped them during childbirth compared with a similar proportion in Nigerian study which showed that 35.7% of women reported slapping during childbirth at health facilities [19]. In this study negligent care was reported by 30.3% of women which is markedly higher than the study conducted in Tanzania with only 7.9% reporting negligent care [20]. Respondents who gave birth by caesarian section were four times more likely to be disrespected and abused than respondents who had gave birth through vaginal delivery perhaps it is due to staff work load and attitude of health care provider. The results of this study show that the prevalence of disrespect and abuse at 43% is lower than that reported in Addis Ababa, Ethiopia where the comparable figure for facility-based childbirth was 76.8% [17].The discrepancy may be due to services improvement. Similarly, the overall prevalence of compassionate and respectful maternity care was 57% which is notably higher than the 21.4% reported in the study conducted in Addis Ababa [17]. The study also revealed that respondents who were residents of rural areas were six times more likely to experience disrespect and abuse than urban residents. Over all in this study the prevalence of discrimination- free care was experienced by only a third of women, with friendly care reported by a quarter. Income level also made a difference, with respondents in this study having a low family monthly income of < 3000 Ethiopian birr being twice as likely to be disrespected and abused as respondents with a higher family monthly income. This finding is in line with the study conducted in Addis Ababa which showed that respondents with low family monthly income were significantly more likely to report disrespect and abuse [17]. Respondents who have no intention to give birth in a health facilities were six time more likely to report disrespect and abuse than respondents who have the intention to give birth in a health facilities. The finding of this study showed that the prevalence of disrespect and abuse is high. To reduce this high prevalence all stakeholders who were working to reduce maternal and child mortality must be involved to promoting respectful maternity care for all child bearing mothers. In addition to this strengthening responsibility through legal redress, citizen involvement on hospital administration boards, client health care charters, improving the quality of the work environment for providers, training and introduction of care standards is very important to promote compassionate and respectful maternity care.

## Conclusions

The results of this study show that the prevalence of disrespect and abuse during childbirth was 43% among the women studied. Abuse of mothers during facility child birth is a health facility failure and a violation of women’s rights as well as an important barrier to women seeking institutional delivery. Independent factors identified that increased disrespect and abuse included low family monthly income, rural residency, complications during delivery, caesarian birth and intention not to use maternity service. Therefore, to reduce disrespect and abuse, attempts to break down barriers between health workers and clients through regular community facility dialogue and strengthening the health system is a vital factor in inviting more women to health facilities and for improving institutional delivery. It is every woman’s right to give birth in a woman centered context with compassionate and respectful care. Provision of woman-centered care in compassionate and a respectful manner needs to be given adequate emphasis to attract more women to health facilities, and to make services more women friendly.

## Abbreviations

AOR: Adjusted odds ratio
CI: Confidence interval
MCHIP: Maternal and child health integrated program
